# What is rural? Examining the relationship between human populations and their inter-connectedness in the context of communicable disease transmission

**DOI:** 10.1186/s12942-026-00456-8

**Published:** 2026-03-14

**Authors:** Cassandra Boutelle, Patrick Corbett, Andrew Gibson, Frederic Lohr, Catherine Swedberg, Jesse Blanton, Ryan Wallace

**Affiliations:** 1 United States Centers for Disease Control and Prevention, Poxvirus and Rabies Branch, 1600 Clifton Ave, Atlanta, GA USA; 2 Mission Rabies, 4 Castle Street, Cranborne, Dorset BH21 5PZ UK

**Keywords:** Urbanicity, Risk map, Communicable disease, Population estimation, Hotspot analysis

## Abstract

**Background:**

Rurality and urbanicity are recognized determinants of public health outcomes that influence policy and resource allocation. However, many commonly used methods to classify the rural-urban continuum, such as the Rural-Urban Commuting Area (RUCA) codes, lack applicability in low and middle-income countries (LMICs). This study introduces the Settlement Type and Road Connectivity (STARC) methodology, which offers a standardized and accessible approach to classifying regions along the rural-urban continuum.

**Methods:**

Leveraging open-source software and readily available data, STARC generates detailed maps composed of small hexagonal polygons. Each hexagon unit is assigned one of twenty-four categorical STARC codes based on its estimated population density and community-based road connectivity. Collectively, the hexagon units comprise the base layer STARC map. Additional metrics related to disease transmission can be overlayed onto each STARC hexagon unit in order to perform hotspot analysis via the local Gi* statistic and create transmission zones. All operations within the STARC process have been packaged into a publicly available tool on GitHub.

**Results:**

To demonstrate the STARC process, we executed the full STARC methodology at the local, national, and regional level for study areas in Central and Western Africa. Free roaming dog densities were selected as the metric of interest in order to identify hotspots and transmission clusters for dog-mediated rabies.

**Conclusions:**

In our analysis we demonstrate that STARC codes can be used to standardize the rural-urban continuum and better understand the distribution of connectedness of populations. Hotspot analysis of free roaming dogs in several African countries shows that dog populations, a key factor in rabies transmission, are often concentrated in urban and peri-urban areas, many of which span domestic and international boundaries. By providing a dynamic and data-driven approach to understanding the rural-urban landscape, STARC offers a valuable tool for public health interventions in LMICs.

**Supplementary Information:**

The online version contains supplementary material available at 10.1186/s12942-026-00456-8.

## Background

Researchers and leading public health institutions have identified rurality and urbanicity as significant determinants of public health outcomes and disparities [1–5]. The complex socioeconomic and geospatial dynamics underlying the rural-urban continuum significantly affects the cost of health interventions, allocation of vital community resources, characterization of mobile populations, and transmission dynamics behind communicable diseases [3, 6–9]. Additionally, classifying an area as urban or rural can result in markedly different policy implications when assessing community needs and efficient utilization of limited public resources [8].

Accurate assessments of the rural-urban continuum are especially important when conducting community-based disease interventions [10–12]. Interventions such as educational programs, vaccination campaigns, and therapeutic delivery are integral to preventing the spread of communicable diseases such as HIV, dengue, tuberculosis, and malaria [6–8, 13]. However, health systems in low and middle income counties (LMICs) often do not have the personnel, vehicles, and medicines to implement community-based programs that offer equitable access and effective disease control for all communities [2, 3]. As such, these health systems must often strategize how to maximize and effectively target intervention efforts within the constraints of limited resources [14]. In order to benefit the most people at the lowest cost, LMICs often prioritize many public services within urban centers at the expense of rural populations [2, 5, 15]. This presents a significant hurdle for public health disease control programs that lack robust international funding, such as those dedicated to neglected tropical diseases (NTDs), as well as high-consequence and low-burden diseases. Moreover, rapid rates of urbanization have also led to growing health inequalities within urban centers where even increased proximity to health services may not be enough to compensate for existing socioeconomic barriers [15].

Many countries have developed binary, geo-political boundaries to designate areas as “urban” or “rural” and these designations are often devoid of consideration as to the density or connectedness of the communities. For example, many anti-rabies dog vaccination campaigns utilize city boundaries to define the extent of campaign efforts, ignoring the often densely populated communities that lie just outside of these geo-political boundaries [16, 17].

Recent studies have illustrated the importance of broadening the understanding of rural and urban as a continuum rather than a binary system [4, 5, 8]. As a result, there has been an increased effort to develop classification systems of rural versus urban in order to better understand the epidemiology of infectious diseases, assess needs when orchestrating community-based interventions, and maximize the effective allocation of limited public resources [1, 4, 5, 8]. However, defining rural versus urban is not necessarily intuitive. For example, a study by Castle et al. found significant discordance between how US pharmacists and the US Census system classified their local communities along the rural-urban continuum [18]. Thus, researchers have noted the importance of developing more standardized and widely adoptable definitions of the rural-urban continuum.

In 1990, the United States Department of Agriculture (USDA) developed the Rural-Urban Commuting Area (RUCA) codes to better characterize communities as well their challenges and needs with respect to delivery of publicly funded programs [5, 8, 19]. Under the RUCA classification system, a combination of population density estimates and work-commuting data is integrated to define census-level regions along a sliding scale of 33 descriptive groups [8, 19]. This more granular system of regional characterization allows policymakers to strategically distribute government, healthcare, and infrastructural resources throughout the United States [8, 19]. Re-envisioning rural and urban as a continuum, rather than binary, addresses gaps in health equity and is an example from which LMICs may benefit [8].

Since the inception of RUCA codes, additional methods have been developed to categorize administrative units along the rural-urban continuum that use publicly available data such as the urban-rural catchment areas (URCA) codes developed by Cattaneo et al. and degree of urbanization (DEGURBA) classification developed by the European Commission [4, 5, 20].

The URCA system adopts concepts from the Central Place Theory (CPT) and classifies localities based on the estimated time it would take to reach the closest or most hierarchically relevant urban center. However, the methodology behind creating RUCA codes can be difficult to replicate in LMICs that lack the extensive census population and commuting data that most advanced approaches require [4, 5, 8]. Moreover, URCA codes lack fine resolution analysis, as is often needed for community-based interventional planning, and rely on the assumption that rural localities operate around one central urban center, which is often not the case.

DEGURBA, which is endorsed by the UN Statistical Commission and widely used, classifies human population density on a 1km^2^ scale, then identifies clusters of human populations as different levels of local administrative units [20]. While this fine resolution is favorable for identifying human population centers, this method does not take into account factors of the built environment, such as road networks.

In an attempt to address limitations with utilizing rural-urban classification systems for community-based disease interventions, we developed the Settlement Type and Road Connectivity (STARC) methodology. In this study, we describe the STARC methodology and demonstrate its utility in public health with the example of rabies control intervention.

## Methods

### Data sources and analysis tools

STARC was developed exclusively with open-source software to ensure that methods are reproducible and accessible to LMICs. R programming language version 4.0.3 was used for statistical analysis and data visualization, incorporating several data processing and spatial R libraries such as tidyverse, rhdx, rgdal, terra, sf, and raster [21–27]. We also used QGIS 3.16.11, an open-source GIS software, to create additional custom maps to help evaluate and explain the processes developed in the STARC tool [28]. The R code underlying the STARC tool as well as some example STARC map outputs can be found on this public-facing CDCgov GitHub page https://github.com/CDCgov/STARC.

National and subnational borders were obtained from the geoBoundaries API at www.geoboundaries.org [29]. OpenStreetMap (OSM) road networks vector layers were downloaded from download.geofabrik.de [30, 31]. Alternative data sources for road networks were explored in Additional File 1. OSM’s global coverage and universal road hierarchy makes it the most viable option for this analysis, despite occasional inconsistencies with the fclass variable and data density. If a more complete data source exists for a specific study area, users should make appropriate changes to the STARC methodology to account for differences in road classification hierarchies.

Hexagon grids for each country were created using Uber’s H3 hexagonal hierarchical geospatial indexing system via the H3jsr R library [32, 33]. We chose to use hexagonal grids due to advantages they have in spatial analysis compared to rectangular grids such as reduced sampling bias due to lower perimeter-to-area ratios, isotropic distances among neighboring grid units, and greater visual clarity in visualizations [34–37]. H3 hexagons were created using resolution value of 8 which corresponds to an average hexagon unit area of around 0.73 km^2^ [33]. Each hexagon will be referred to as an individual hexagon unit and has a unique H3 index.

We obtained country-wide population estimates at the 1 × 1 arcsecond resolution (around 30 × 30 m at the equator) from Meta’s High Resolution Settlement Layer (HRSL) [38–40]. Meta’s HRSL combines machine learning models with modern high-resolution satellite imagery and available census data to estimate human population at the level of individual buildings [39–41]. We pulled HRSLs utilizing the Humanitarian Data Exchange (HDX) API via the rhdx R library [22].

We used Meta’s HRSL data for our analyses due to ease of use, spatial resolution, and evidence of data quality advantages. Studies suggest that HRSL is a reliable source of population estimates and has a relatively high agreement with established sources such as census data and WorldPop estimates in various regions [40–42]. Additionally, analysis performed by Meta indicates that its HRSL may be beneficial when estimating population in rural regions [40]. However, there are various data sources for human population estimation that may be adapted to the STARC process and may be more appropriate given the specific region or country in question including Gridded Population of the World (GPW), Global Rural Urban Mapping Project (GRUMP), Global Human Settlement Layer-Population (GHS-POP), or LandScan Population [42]. The methods presented in this manuscript and the associated repository code are designed for use with HRSL. If users elect to implement STARC methodology with other population sources, the methods should be adjusted to account for differences in scale and other spatial characteristics, as these drastically impact population density estimates.


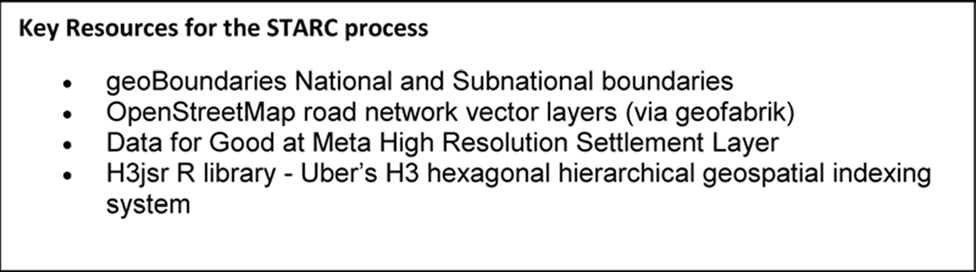


### Spatial processing: standardizing the coordinate reference system (CRS)

To standardize assigning projections to our raster, vector, and polygon layers we utilized the suggest_crs function in the crsuggest R library [43]. This function provides suggestions for the projected coordinate reference systems to use in CRS transformations based on a spatial object input. We used the national border obtained from the geoBoundaries API, which has a default geographic CRS of WGS 84, as the input in the suggest_crs function [29]. We filtered the suggested coordinate reference systems from the suggest_crs function down to the most recommended UTM zone projected CRS. The projected CRS was saved as a global variable and all spatial objects used throughout the analysis for the specified study were subsequently projected using this CRS. The st_transform function within the sf R library was used to transform polygon and vector objects while the projectRaster function in the raster R library was used to reproject raster objects [25–27].

### Final products

The STARC process results in two products: (A) a STARC map and (B) a risk map that shows disease hotspots and transmission clusters. The STARC map is composed of hexagonal grid, each hexagon unit containing a STARC code corresponding to its population density and road connectivity. This first product serves as a basemap that can be used to both visualize the rural-urban continuum and to construct more epidemiologically-specific outputs.

The second product is a map with epidemiologically-relevant disease hotspots and transmission clusters built based off of a continuous variable contained in each hexagon unit within the STARC map. This continuous variable should be relevant to a certain disease’s transmission dynamics or host prevalence, depending on the goal of the analysis. For example, we performed disease hotspot and transmission cluster analyses using estimated free roaming dog densities, as the authors’ primary responsibility is rabies control and free roaming dog density is a suitable indicator of rabies transmission risk.

To fully demonstrate the uses of the STARC process, we conducted analyses on various scales: locally, (focused on 1 city, N’Djamena, Chad), nationally (4 individual countries in West Africa), and regionally (4 countries in West Africa analyzed as one study area). The following methodology presents the steps to produce each product. A flowchart detailing the classification of STARC codes is included in Additional File 2. The STARC tool performs these steps automatically once a particular study area has been selected and input parameter values are identified.

### (A) Classification: STARC map

#### Settlement type

##### Population density

The STARC tool was designed to download the relevant administrative border files, human population rasters, and OSM road layers for each specified country [29, 30, 33, 39]. An H3 hexagonal layer is subsequently generated across the extent of the study area that was buffered by 100 m to prevent gaps along the outer edges of the border. Hexagon units that extend beyond the desired borders were clipped using the sf function st_crop(). To estimate the population within each hexagon using the raster population file, the exact_extract() function within the exactextractr R library was used to proportionally divide the population encoded in each raster cell according to the hexagon units they intersected [38]. For example, a raster cell in a 40/60 split between two different hexagon units would contribute 40% and 60% of its total population to the hexagon unit values, respectively. The proportional population values were aggregated within each hexagon unit and the population density for each hexagon unit was calculated by dividing the total estimated population by the total area of the hexagon unit.

##### Settlement Type Code

We defined a Settlement Type Code (STC) according to six settlement categories, S1 to S6, based on the population density within each hexagon unit. Population cutoffs for these categories were selected based on global population density distributions and informed by existing urban classification schemes [44]. A logarithmic scale was used to define hexagon units as very high density (S1, >5000 people/km^2^), high density (S2, 500-4999 people/km^2^), dense (S3, 50-499 people/km^2^), low density (S4, 5-49 people/km^2^), very low density (S5, 1-4 people/km^2^), and zero (S6, 0 people/km^2^).

#### Road connectivity

##### Road network levels

A road classification was defined based on OSM road layer attributes. OSM categorically defines each road vector according to its feature class, encoded in the variable “fclass” [30]. These feature classes were grouped to define the primary, secondary, and tertiary road network levels according to the following list:

● **Primary:** including all values equal to Motorway, Motorway Link, Primary, Primary Link, Trunk, Trunk Link

● **Secondary:** including all values equal to Secondary, Secondary Link, Tertiary, Tertiary Link

● **Tertiary:** including all values equal to Bridleway, Cycleway, Footway, Living Street, Path, Pedestrian, Service, Steps, Track, Track grades 1-5, Unclassified, Unknown

● **Unconnected:** all areas with no road connection

##### Community polygon analysis

Studies show that the organization of road infrastructure tends to shape communities by clustering people around accessible routes, especially in metropolitan and suburban areas, creating a reliance on common road systems [64] . Additionally, rural transportation studies underscore that close proximity to roads significantly influences connectivity in low- and middle-income areas, reinforcing the role of nearby roads in shaping accessible transportation networks for local populations [65]. To ensure that all hexagon units that are part of a single community are classified as the same road network level, human population raster cells were buffered by a distance of three cells horizontally and vertically (roughly 90 m) using the buffer() function within the terra R library [24]. The buffered raster cells that intersected each other were subsequently merged and converted into individual polygons, each of which represent a “community.” These community polygons were hierarchically assigned a road network level as described above based on the highest value of road types that they intersected. For example, if a community polygon had secondary and tertiary-level roads running through it, the polygon’s road connectivity level would be classified as secondary.

##### Road connectivity code

The Road Connectivity Code (RCC) for each hexagon unit was determined in a stepwise approach. Hexagon units were first hierarchically classified as primary, secondary, or tertiary based on the level of the community polygon they intersected. Then, for those hexagon units that did not intersect community polygons, the RCC was hierarchically categorized by the level of road vectors directly intersecting the hexagon. Hexagon units that did not intersect roads (directly or as part of a community polygon) were classified as disconnected. The final RCCs were then assigned as primary (1), secondary (2), tertiary (3), and disconnected (4).

#### Merging variables

**Fig. 1:**
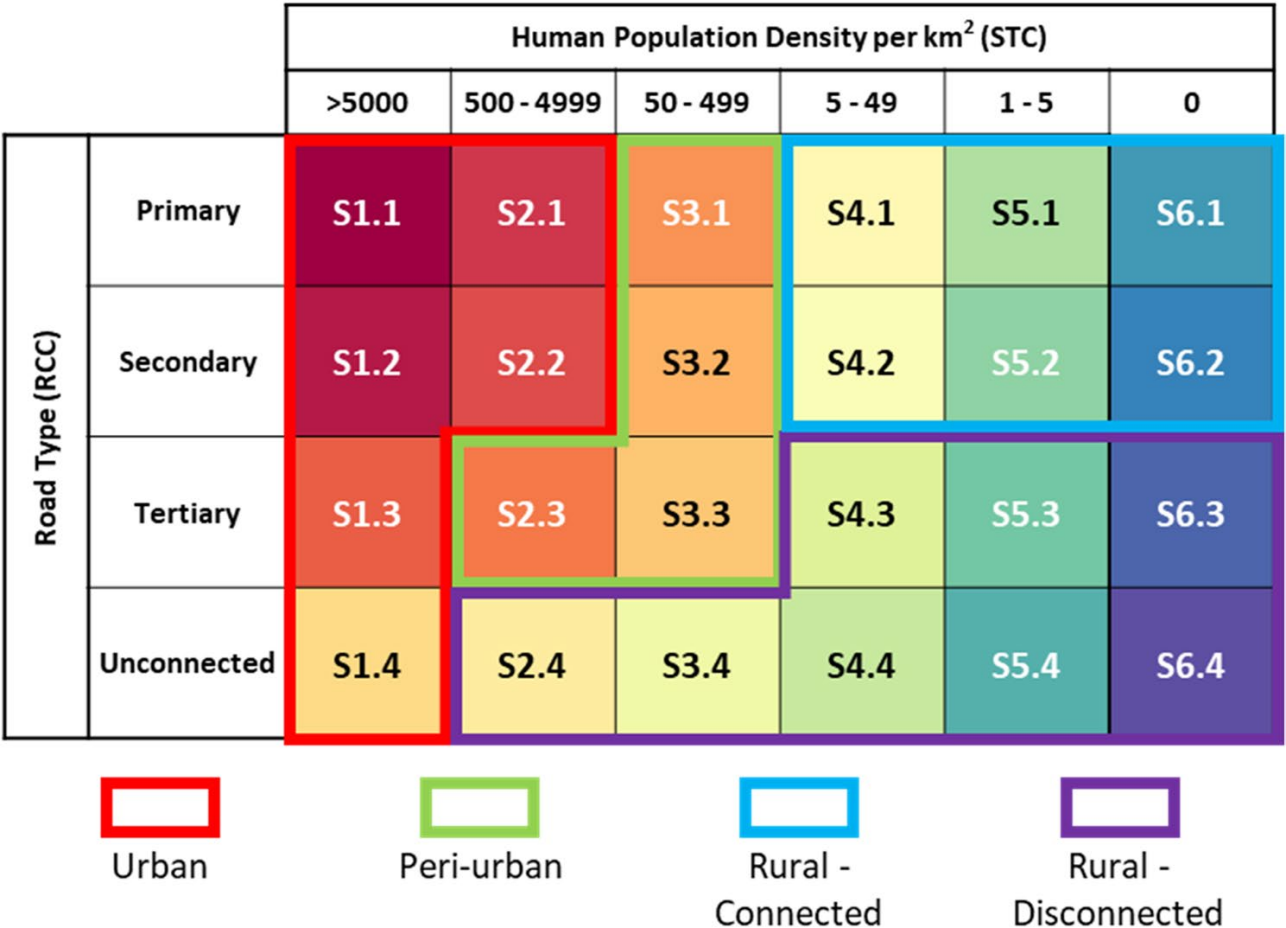
Settlement Type codes (STCs) and Road Connectivity codes (RCCs) are combined to create the final STARC codes. Column labels indicate the population density cut offs for STC and the row labels represent the different RCC categories. The colored outlines indicate the STARC codes used to define urbanicity in our analysis: “Urban” (red) was defined as 1.1, 1.2, 1.3, 1.4, 2.1, and 2.2. “Peri-Urban” (green) was defined as 2.3, 3.1, 3.2, and 3.3. “Rural – connected” (blue) was defined as 4.1, 4.2, 5.1, 5.2, 6.1, and 6.2. “Rural – disconnected” (purple) was defined as 2.4, 3.4, 4.3, 4.4, 5.3, 5.4, 6.3, and 6.4. The color of each cell represents the color of each STARC code as depicted on STARC maps.

The STCs and RCCs were combined to create the STARC codes in the format S*x.y* where *x* is the STC and *y* is the RCC (Fig. 1). A brief comparison of STARC code classification to other established rural-urban classification schemas can be found in Additional File 1. In recognition that not every mapping exercise will require the full specificity of all 24 STARC codes, and that interpretation of a map with a 24-color scale may be difficult, we present examples of alternative color scales in Additional File 3.

### (B) Risk identification: disease hotspots and transmission clusters

#### Hotspot analysis

To identify epidemiologically significant disease hotspots within the STARC map, a continuous variable related to host or transmission dynamics must be identified. In our case, we used free roaming dog population density and the variable of interest that most impacts rabies transmission. Dog population density was calculated using human-to-dog ratios gathered from published literature (Additional File 4).

The hotspot analysis was performed by calculating the standardized Getis-Ord *G*_i_* statistic for each hexagon unit [45, 46]. Statistical significance was determined by applying Bonferroni correction to the p-value to account for multiple comparisons [45–47]. The *G*_i_* statistic for a particular hexagon unit *i* was a ratio of the local sum of nearby hexagon unit values compared to the sum of all hexagon units within the study area (e.g., country or region).

Equation 1 shows how the *G*_i_* statistic was calculated where *n* was the total number of hexagon units, *x*_*j*_ was the attribute value for all hexagon units *j* including hexagon unit *i*, and *w*_*i, j*_ was the spatial weight between hexagon unit *i* and all neighboring hexagon units *j*. Spatial weights *w*_*i, j*_ were assigned a uniform value of one for all neighbor pairs and a value of 0 for all non-neighbor pairs.

1 $$\:\begin{array}{c}{G}_{i}^{*}=\frac{{\sum\:}_{j=1}^{n}{w}_{i,j}{*x}_{j}}{{\sum\:}_{j=1}^{n}{x}_{j}}\end{array}$$

Under the assumption of complete spatial randomness, the expected value for *G*_i_*, *E(G*_i_*), is described in Eq. 2. Since we used a uniform spatial weight value of one for all neighbors, the sum of *w*_*i, j*_ was the number of hexagon units defined as neighbors of hexagon unit *i*, including hexagon unit *i* itself.

2 $$\:\begin{array}{c}E\left({G}_{i}^{*}\right)=\frac{{\sum\:}_{j=1}^{n}{w}_{i,j}}{n}\end{array}$$

When conducting the Getis-Ord *G*_i_* test to identify hotspots of high values, the null hypothesis was that *G*_i_* is equal to *E(G*_i_*), and the alternative hypothesis was that Gi* is greater than its expected value.

$$\:{H}_{0}:\:{G}_{i}^{*}=E\left({G}_{i}^{*}\right)$$

$$\:{H}_{1}:\:{G}_{i}^{*}>E\left({G}_{i}^{*}\right)$$

The calculations for the variance of *G*_i_*, *V(G*_i_*), are shown in Eqs. 3–5 where $\overline{X}{}$ and *S* are the mean and standard deviation of all observations in the analysis, respectively. Equation 6 depicts the formula for the standardized *G*_i_* statistic, *Z(G*_i_^*^), which is also the z-score of *G*_i_*. Resulting p-scores were derived according to the normal distribution function. P-scores were then adjusted using Bonferroni correction to account for multiple comparisons, as shown in Eq. 7 where *n* is the number of hexagon units in the study area. Any hexagon units with values above the global mean (Eq. 3) and Bonferroni-corrected p-scores lower than 0.005 were considered statistically significant hotspots of high values that rejected the null hypothesis of spatial randomness.

3 $$\:\begin{array}{c}E\left({G}_{i}^{*}\right)=\frac{{\sum\:}_{j=1}^{n}{w}_{i,j}}{n}\end{array}$$

4 $$\:\begin{array}{c}\:S=\sqrt{\frac{{\sum\:}_{j=1}^{n}{{x}_{j}}^{2}}{n}-{\stackrel{-}{X}}^{2}}\end{array}$$

5 $$\:\begin{array}{c}V\left({G}_{i}^{*}\right)=\frac{n{\sum\:}_{j=1}^{n}{w}_{i,j}^{2}-{({\sum\:}_{j=1}^{n}{w}_{i,j})}^{2}}{{n}^{2}\left(n-1\right)}*\:{\left(\frac{S}{\stackrel{-}{X}}\right)}^{2}\end{array}$$

6 $$\:\begin{array}{c}Z\left({G}_{i}^{*}\right)=\frac{{G}_{i}^{*}-E\left({G}_{i}^{*}\right)}{\sqrt{V\left({G}_{i}^{*}\right)}}\end{array}$$

7 $$\:\begin{array}{c}Adjusted\:P\left({{G}_{i}^{*}>E\left({G}_{i}^{*}\right))=\:P({G}_{i}^{*}>\:E(G}_{i}^{*}\right))*n\end{array}$$

Research suggests the extended home range (HR) size for free roaming dogs spans 0.9–40.5 ha, or around a 50–360-meter radius [48]. We chose to liberally define neighbors using a search radius of 1.7 km so that hexagon units *j* with centroids 1.3 km from the outer edge of hexagon unit *i* were considered neighbors and were factored into hexagon unit *i*’s local sum calculation. See Additional File 5 for an illustration of how neighboring hexagon units were identified using a search radius.

Neighbors were identified using the dnearneigh() function in the spdep R library [49]. We assigned each hexagon unit as its own neighbor using the spdep::include.self() function and assigned a spatial weight of one for all neighbor pairs using the spdep::nb2listw() function. Calculations for *G*_i_^*^, *E(G*_i_^*^), and *Z(G*_i_^*^) were performed by the spdep::localG() function. Bonferroni corrected p-values were determined using the stats::p.adjust() function and hotspots with statistically significant high values were identified using the spdep::hotspot() function.

#### Creating transmission clusters

Disease hotspots, identified according to the above methodology, were used as seeds around which transmission clusters were formulated. Transmission clusters are larger and more cohesive than hotspots, and they were intended to highlight areas of epidemiological or ecological importance based on the proximity of hotspots to each other and to other hexagon units with the variable of interest (e.g., free roaming dogs).

To create the transmission clusters, a list of STARC codes to be included in defining the cluster extent was defined. For our purposes, we decided to use STARC codes S1.1 - S3.4, which mostly resemble urban and peri-urban environments. All hexagon units with a STARC code in this list that were not defined as part of a hotspot were buffered by a predefined distance, using sf::st_buffer(). Buffered hexagons that intersected with each other were joined into individual polygons using the sf::st_union() function. The buffer size should be epidemiologically pertinent to the transmission or host dynamics of interest. Given the limited ecological range of free roaming dogs observed in multiple studies, we defined a buffer size of 500 m such that appropriately coded hexagon units within 1 km of each other were included within a single cluster [48, 50].

Similarly, all hexagon units in hotspots, regardless of their STARC codes, were buffered by the same user-defined size in meters. The buffered hotspots were then joined with the grouped polygons (created in the previous step) to create the final cluster boundaries. Any polygons that do not intersect with the buffered hotspots were not included in the final cluster boundaries. All hexagon units completely contained within the cluster boundaries, regardless of STARC code, were included in the final clusters).

#### Clustering summary statistics

As part of the STARC tool, summary statistics are generated for each transmission cluster including the total area in km^2^, number of hotspots contained, estimated population and population densities, and political regions intersected. Additionally, clusters are ranked in priority based on the relative population contained within their associated hotspots, thus defining clusters by the total number of hotspots and their respective population densities. Similar summary statistics are created for each hotspot and for the study area as a whole (Table 1).

### Regional STARC analysis

The STARC process was broadened to also include multi-national, or regional, outputs. This process included generating STARC maps for each country in the region separately. The spatial data is then compiled to generate a hotspot and cluster analysis for all countries together. Performing regional STARC analysis in this manner allows for hotspots and potential transmission clusters to be identified without the artificial barrier of national political borders. The STARC maps include the STARC classification across the entire country, national and regional borders, as well as primary and secondary roads. Cluster maps include political borders, road layers, hotspots, and transmission clusters.

For this manuscript, we ran individual country analyses and a regional analysis for four West African countries (Guinea, Sierra Leone, Liberia, and Côte d’Ivoire) as well as a rabies cluster analysis for N’Djamena, Chad, a well-documented rabies control program, and the surrounding area including Kousseri, Cameroon. Complete analyses were run individually for each country using HDRs estimated from published literature (Additional File 4) [16, 51–54].

### STARC run-times

**Fig. 2:**
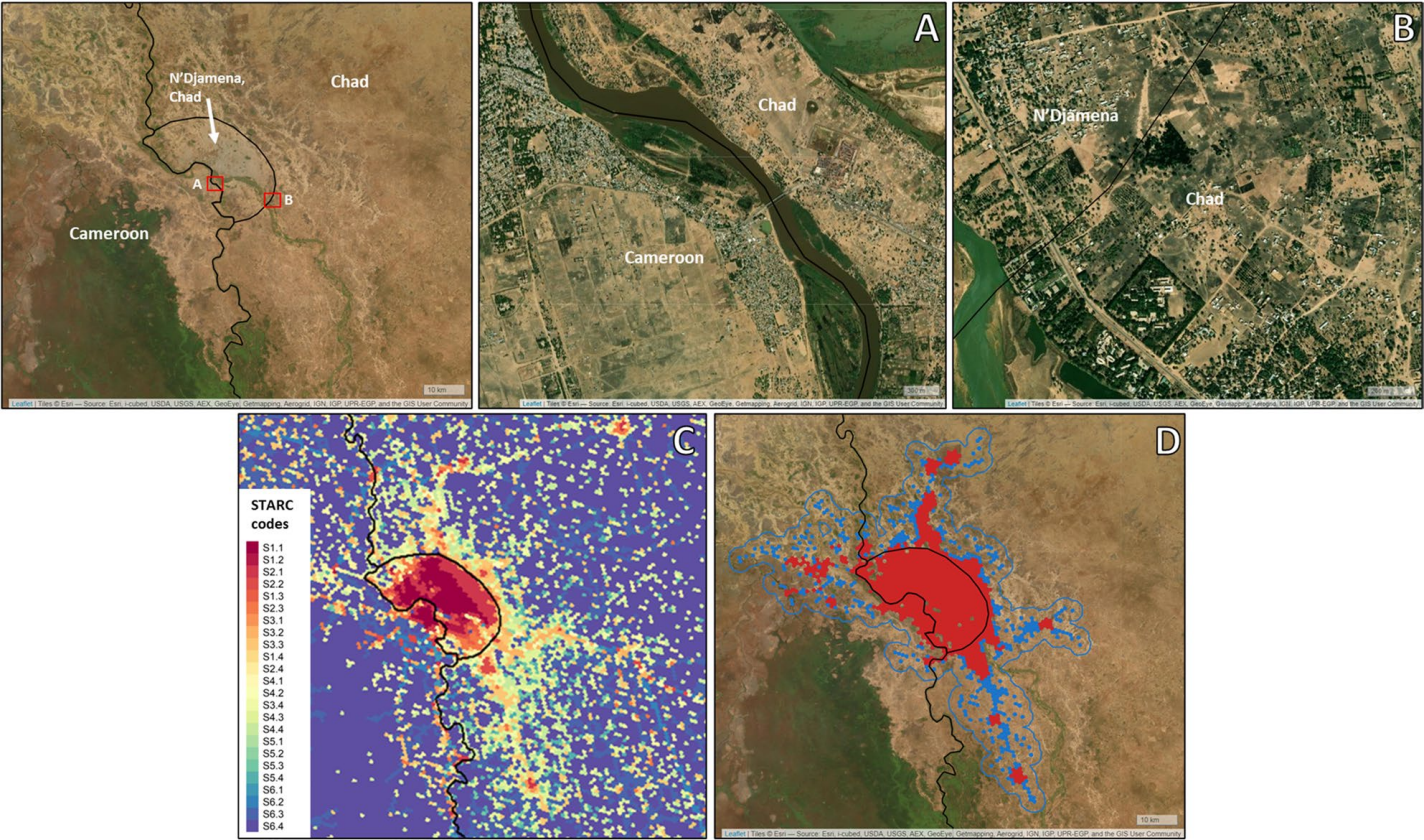
STARC and disease hotspot and transmission cluster maps, for the area surrounding N’Djamena, Chad. Black outline depicts the N’Djamena and national administrative boundaries. Panel [**A**]: Satellite imagery demonstrating the close proximity of dense human populations separated by an international border and river. Panel [**B**]: Satellite imagery demonstrating the continuous human habitations and road networks across the N’Djamena city administrative boundary. Panel [**C**]: STARC Map of N’Djamena, Chad and surrounding area. Panel [**D**]: Transmission Cluster, demonstrating likely dog-maintained rabies virus transmission occurring across international and N’Djamena political borders. Satellite imagery from Esri obtained via leaflet.providers R library [55, 56]

**Fig. 3:**
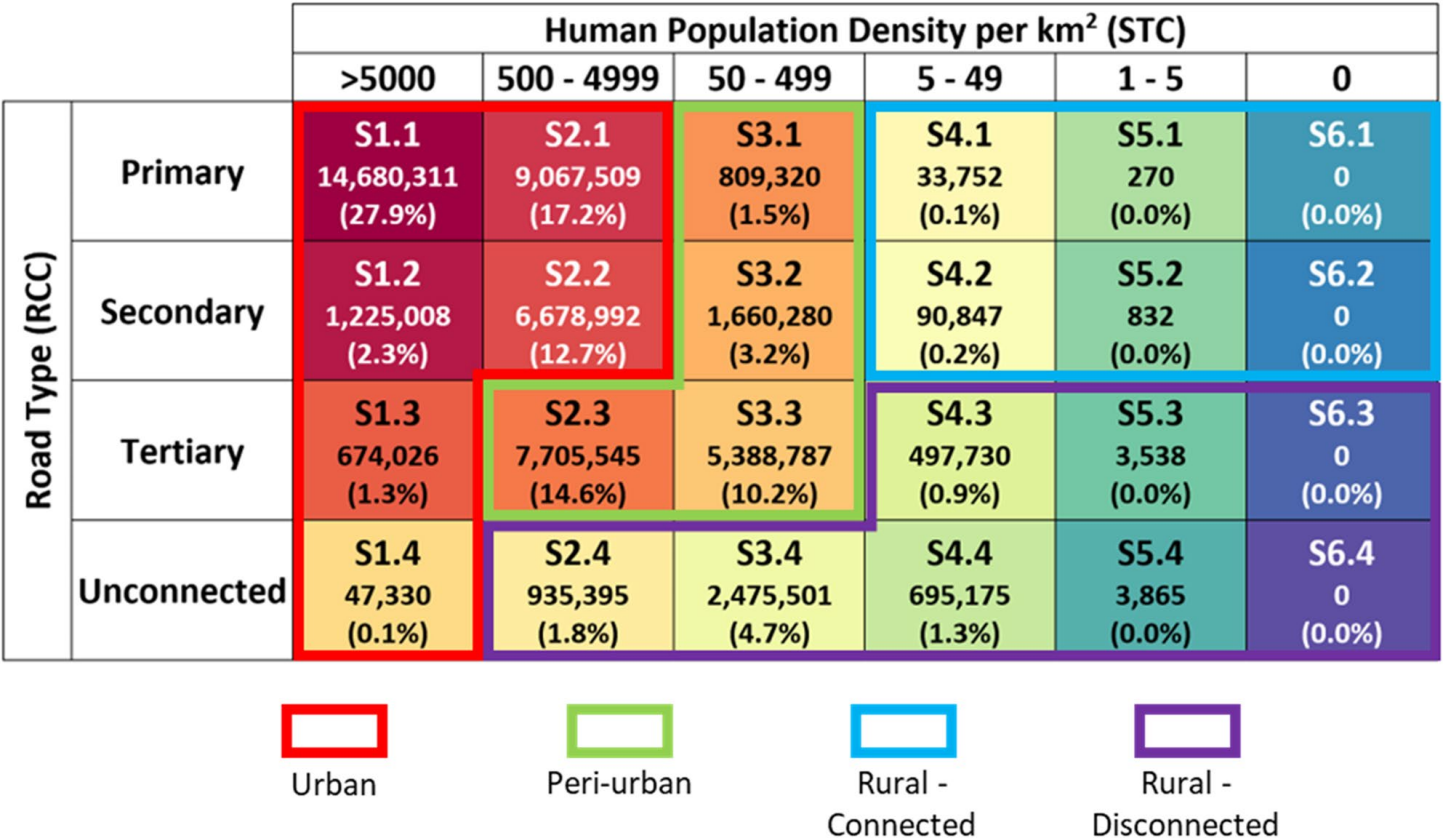
Total and percent population for each STARC category within our particular study area in West Africa. Each cell in the figure contains the STARC code as well the total and percent of the estimated population contained within hexagon units of the same STARC code

**Fig. 4:**
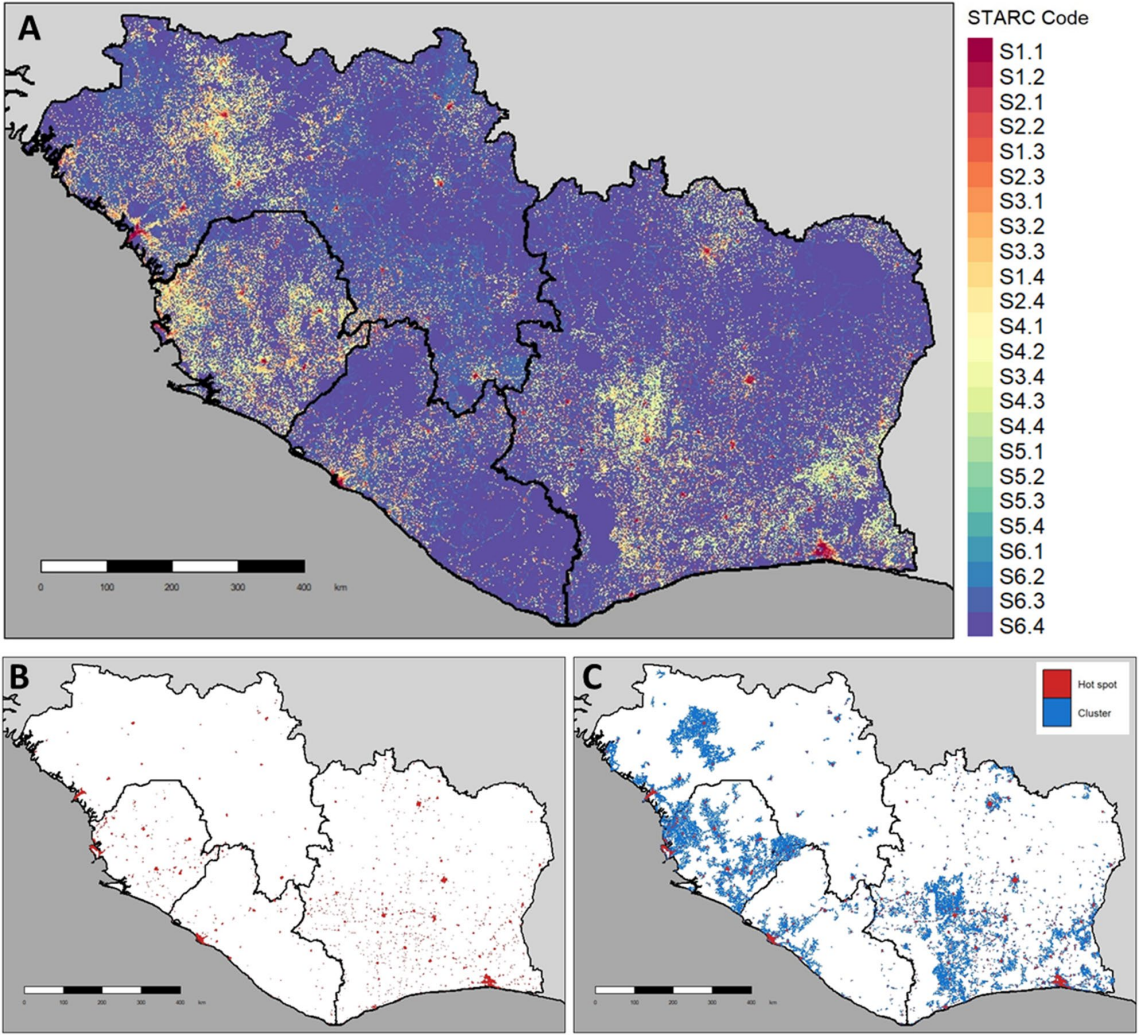
West Africa regional STARC analysis depicting the distribution of people and road networks across four countries: Guinea, Sierra Leone, Liberia, and Côte d’Ivoire. Panel [**A**] depicts the STARC codes. Panel [**B**] depicts the free roaming dog hotspots in red, which where were used as a proxy indicator of dog-maintained rabies virus. Panel [**C**] depicts the rabies hotspots (red) and transmission clusters (blue)

Generating a national STARC analysis requires manual selection of parameters in a R shiny application including the country, the *G*_i_^*^ neighbor search radius, the STARC codes to include in the cluster analysis, and buffer distance to use when identifying clusters. Running the full STARC analysis can be computationally demanding, particularly when constructing the H3 hexagonal grid, aggregating raster information into hexagonal units, and calculating the standardized Gi^*^ statistic for each unit. Run times were recorded for the STARC maps produced for this analysis (Figs. 2, 3,4).

**Table 1:** Comparison of STARC-derived characteristics across four West African countries and N’Djamena, Chad

Total Area (km^2^)	Guinea	Sierra Leone	Liberia	Cote d’Ivoire	Individual: West Africa**	Regional: West Africa**	*N*’Djamena, Chad Cluster
	246,852	73,741	96,888	324,575	742,056		416***
**Hotspot Area (km** ^**2**^ **) and Number**	1,975 (*n* = 146)	1,461 (*n* = 79)	1,391 (*n* = 147)	5,548 (*n* = 698)	10,375 (*n* = 1,070)	11,153 (*n* = 1,308)	794 (*n* = 10)
**Cluster Area (km** ^**2**^ **) and Number**	13,382 (*n* = 115)	10,841 (*n* = 19)	4,004 (*n* = 101)	19,982 (*n* = 539)	48,209 (*n* = 774)	48,569 (*n* = 877)	1,136 (*n* = 1)
**Total Human Population**	13,161,807	7,991,351	5,092,025	26,428,829	52,674,012		1,848,318***
**Hotspot Human Population**	6,469,004	3,742,216	3,000,283	15,189,541	28,401,044	28,699,598	2,209,917
**Cluster Human Population**	8,838,867	6,197,190	3,690,677	19,241,032	37,967,766	38,214,734	2,269,228
Proportion of total population residing in Urban settings*	56.9%	56.7%	62.2%	65.0%	61.5%		87.2%
Proportion of total population residing in Peri-Urban settings*	31.8%	36.7%	30.7%	26.1%	29.5%		9.2%
Proportion of total population residing in Rural settings*	11.3%	6.6%	7.1%	8.9%	9.0%		3.6%
**Total Estimated Free Roaming Dog Population**	382,173	945,951	544,011	2,746,863	4,618,998		52,000***
**Hotspot Free Roaming Dog Population**	171,027	273,550	231,699	1,136,454	1,812,730	1,939,683	61,475
**Cluster Free Roaming Dog Population**	246,472	639,436	336,236	1,704,649	2,926,793	3,054,973	64,187

*For the purposes of this analysis, STARC Codes 1.1, 1.2. 1.3, 1.4, 2.1, and 2.2 were considered “Urban”. STARC Codes 2.3, 3.1, 3.2, and 3.3 were considered “Peri-Urban”. All other STARC codes were considered “Rural”

** “Individual: West Africa” sums analyses from each country performed separately; “Regional: West Africa” performs hotspot and cluster analysis for all countries at once

*** This represents the estimated value within the N’Djamena city boundary, presented for comparison to the estimated values within the entire transmission cluster

## Results

The final outputs of the STARC process for each analysis area STARC map (similar to Figs. 2C and 4A) and a risk map (similar to Figs. 2D and 4C). The comparison analysis, located in Additional File 1, shows that STARC code classification is loosely aligned with DEGURBA clusters. STARC’s population density classification aligns with the DEGURBA cluster level, and the inclusion of STARC’s road connectivity classification creates additional level of stratification that is not accounted for by DEGURBA’s clusters. Road connectivity classification makes STARC classification uniquely suited for the accompanying disease hotspot and cluster analyses, especially for those diseases with transmission facilitated by road networks. Summary statistics from the hotspot and cluster analysis are exported to a Microsoft Excel file. On a standard laptop computer (Intel^®^ Core™ i7-8665U CPU @ 1.90gHz,2112 Mhz, 4 Cores, 8 Logical Processors, 16127 MB total RAM), the average processing time for an individual country’s STARC map was about 3 h, with variation dependent the country’s size.

### Local study area: N’Djamena

A STARC analysis was run for the area surrounding N’Djamena, Chad which borders Kousseri, Cameroon. Figure 2C illustrates the output of the initial STARC map, and Fig. 2D visualizes the risk map made up of epidemiologically-relevant hotspots and clusters, based on estimated free roaming dog densities. When considering the transmission cluster that encompasses N’Djamena, Chad and Kousseri, Cameroon, 64,187 free roaming dogs are estimated to reside within the cluster; 52,000 of these dogs reside within the N’Djamena city boundary (81.0% of the rabies at-risk dog population) (Table 1). This transmission cluster encompasses a 1,136 km^2^area, comprised of 10 rabies hotspots located across both countries and extending outside of the city boundary (Fig. 2).

### National and regional study areas: West Africa

STARC analyses were run twice for the West African countries of Guinea, Liberia, Sierra Leone, and Côte d’Ivoire; once for each country individually, and again with all countries at once. The resulting maps of the regional analysis are displayed in Fig. 4. Overall, 61.5% of the West Africa population is expected to reside in an “Urban” community, compared to just 29.5% in “Peri-Urban” and 9.0% in “Rural” (Fig. 3). Differences between countries were noted, with the most urbanization in Cote d’Ivoire (65.0%) and lowest urbanized population identified in Sierra Leone (56.7%). Guinea had the highest rural population (11.3%). Among the four West African countries, the largest segment of the population is found in well-connected (RCC 1) communities and dense communities (STC 2) (Fig. 3). Over 35% of the population of this region resides in poorly connected communities (RCC 3 and 4). Much of the population resides in a S1.1 community (27.9%), followed by S2.1 (17.2%), S2.3 (14.6%), and S2.2 (12.7%).

For the West African countries, 1,308 disease hotspots were identified across a cumulative area of 11,153 km^2^ with an average hotspot size of 8.5 km. Over 28 million people, around 54.5% of the total regional population, were found to reside in potential rabies hotspots. These hotspots are located across 877 transmission clusters covering an area of 48,569 km^2^; just 6.5% of the overall land mass of the region. Over 38 million people (72.5% of the overall population) reside in transmission clusters across these four West African countries. Sierra Leone has the highest proportion of human population residing in a rabies transmission cluster (77.5%). The regional analysis identified an additional 238 rabies hotspots and 103 transmission clusters compared to the individual country analyses. Over 5.5 million people reside within 12 clusters that cross international borders, highlighting the importance of collaborative interventions to effectively control the spread of disease.

There are an estimated 4,618,998 free roaming dogs in the four West African countries; however, only 1.9 million (41%) and 3.0 million (65%) dogs are located within hotspots and transmission clusters, respectively. Nearly 16% of all free roaming dogs are found in rural-disconnected communities, which may influence understanding of disease transmission dynamics and represent a significant barrier to the delivery of veterinary services. Conversely, 44.6% of free roaming dogs are found in urban communities, which may represent a large proportion of dogs more easily reached by veterinary services.

## Discussion

Improving healthcare access and ending epidemics of neglected diseases are primary objectives of the United Nation’s Sustainable Development Goal [57]. Achieving either of these goals requires a strong understanding of the populations at risk. The compounding factors of poverty and rural-dwelling are well-documented barriers to achieving the UN’s goals, yet no standard definition of the rural-urban continuum currently exists in LMICs, especially within the context of epidemiological interventions. The lack of such a standard complicates the comparison of interventional studies, confounds the scientific understanding of disease transmission dynamics, and limits the monitoring and evaluation of healthcare access improvement goals. As an example, rabies is often described as a disease in rural communities, however, STARC analysis of West Africa shows that the majority of dog populations, and the highest densities of dogs, are found in primarily urban and peri-urban settings. Given that dogs in urban communities can typically be immunized against rabies for USD $2.00 versus USD $7.00 in rural areas, using STARC to prioritize interventions in source urban populations can improve the cost effectiveness of vaccination campaigns [58].

STARC is rooted in the methodology of the United States’ RUCA codes, which have been used for decades to allocate public resources, equitably, across the rural-urban continuum. While RUCA was the impetus for developing STARC, RUCA methodology requires data elements that are not commonly available, nor updated, in LMICs. STARC’s methodology, software requirements, and open-source data inputs were all developed with the intention to be accessible to LMICs and easy to update frequently. Additionally, while dependent upon computer processing and the size of the area being mapped, the STARC tool also allows for a user-friendly and relatively time-efficient method to recreate the resulting maps and analyses. Given the highly mobile human populations of many LMICs, due to political instability or inevitable shift towards urban-dwelling for economic opportunities, a tool that can be easily updated to reflect an ever-changing landscape is critical for informing health interventions.

Characteristics that distinguish an area as stereotypically rural or urban, such as level of infrastructure, access to healthcare, living conditions, and economic security all impact the ability to successfully orchestrate communicable disease interventions [6, 7, 59]. Studies have shown that factors such as population density and connectedness influence infectious disease transmission and are integral to understanding the logistics, cost, and reach of interventional strategies [60, 61]. Large-scale public health interventions in LMICs have historically faced difficulties effectively allocating resources across the rural-urban continuum, as documented in previous campaigns on diseases such as Guinea worm disease and polio [6, 7, 59]. A primary goal of developing the STARC method was to inform disease-specific programs on how to best allocate resources beyond the traditional geo-political boundaries, which lack consideration for disease transmission dynamics. Therefore, the process of identifying disease hotspots and transmission clusters becomes a novel aspect of STARC that could be applicable to any disease program facing challenges with identifying populations that would most benefit from public health interventions. While this analysis featured the incorporation of free roaming dog populations as the focus for hotspot and cluster identification, this process would be equally informative for pathogens that are found in other animal species or even human-mediated communicable diseases. Based on the regional analysis, a promising finding for rabies control efforts in West Africa is that roughly 35% of dogs reside in disconnected communities that likely lack the dog densities to maintain rabies. With this information, goals of improving access to both human and dog vaccines may become more feasible and cost-effective.

Dog vaccination programs are commonly implemented at the geo-political level. However, dogs often do not respect the largely invisible boundaries of cities, towns, districts, or even international boundaries. When dog vaccination programs are not well coordinated with neighboring communities, rabies is readily re-introduced into those communities that vaccinate, from neighboring communities that do not.

One such example of this was well documented in N’Djamena, Chad, where a multi-year, high-coverage dog vaccination program was enacted from 2012 to 2014 [16]. While evidence suggested that dog-mediated rabies transmission was halted in the city, cases returned in less than one year. When viewed from the perspective of a STARC analysis, it becomes clear that free roaming dog populations extend beyond the city boundaries. Even the international boundary with Cameroon is a river that is reduced to stagnant pools of water in the dry season and unlikely to be a barrier to disease transmission networks. While 70% vaccination coverage may have been achieved within the city, this coverage drops below the critical threshold for elimination to just 55% when considering the entire STARC-derived transmission cluster, suggesting that transmission was never halted within the full transmission zone. Additionally, geographically-selective vaccination methods that result in pockets of low coverage have been shown ineffective at eliminating dog-mediated rabies [17, 62]. The selection of an appropriate study area is key to ensuring that STARC will identify the complete extent of target populations. These populations can span national boundaries as well as subnational boundaries, as demonstrated by the West Africa and N’Djamena analyses. STARC enables vaccination program managers to better understand how their target populations are connected, irrespective of geo-political boundaries, and can be used to encourage all communities in a common transmission zone to coordinate their vaccination efforts.

While the authors elected to use Meta’s HRSL data for human population estimations and OpenStreetMap for road networks, there are multiple other sources that might better serve users of STARC depending on desired resolution size, acceptable assumptions in underlying population estimation methodology, completeness of road network data, and specific region of interest. Continuous re-evaluation of the ideal global, regional, and national data resources should be conducted and data sources that meet the needs of the analysis should always be prioritized. Additionally, users of the STARC tool must consider the epidemiologic and statistical implications of the data elements that are intended to be modified by the user. For example, choosing different hotspot search radius and cluster buffering distances can significantly alter the final product. While we opted to use 1.7 km and 500 m for these values, respectively, different distances may be more appropriate based on the host or transmission dynamics of interest. Additionally, while we used the same distances for all countries in the paper, it may not be feasible to accurately compare STARC analyses for regions with different distance parameters.

The current STARC classification algorithm does not incorporate distance, either in units of time or meters, an area is from an urban center, which has been found to be an effective predictor indicator of rural versus urban [4, 5]. One particular advantage of STARC is that alternative rural-urban schemas can be applied at the individual hexagon unit level. Thus, the general concept of proximity to urban centers can be incorporated into STARC by evaluating the proximity of each hexagon to a S1 hexagon unit or to clusters of S1 hexagon units. Future work in STARC methodology will focus on incorporating alternative schemas for a multi-layered approach to rural-urban continuum inference. This in turn would allow for multi-variate approximations of variables such as free roaming dog population density.

Much of the global community has moved away from the idea of a binary “rural vs urban” definition, instead opting for more nuanced continuum approaches to characterizing communities. STARC embraces this much needed shift but differs from existing approaches by offering a methodology that is designed for use in LMICs and is easily repeated to account for changing population dynamics. STARC was designed not as a disease-specific tool, but as a method by which disease programs can better understand the distribution and connectedness of their target populations. As demonstrated here, the task of eliminating rabies in West Africa becomes more feasible when viewed through the lens of a STARC analysis; the target dog populations can be clearly visualized, the number of vaccines needed can be reduced, and limited resources can be focused on the communities that are driving transmission. STARC also provides a visual and quantitative rationale to move away from geo-politically defined disease interventions, and to focus on contiguous at-risk populations, irrespective of sub-national and national boundaries. People, their pets, and their diseases all exist across a continuum of population densities and inter-connectedness. Having a standardized method for describing this continuum may help improve global health and propel programs towards successful international goals.

## Supplementary Information

Additional File 1

Additional File 2

Additional File 3

Additional File 4

Additional File 5

## Data Availability

R code behind STARC can be found here on CDCgov’s GitHub page [https://github.com/CDCgov/STARC](https://github.com/CDCgov/STARC). Data for national and regional borders can be found at [www.geoboundaries.org](www.geoboundaries.org). Data for road classifications can be found at download.geofabrik.de. The H3jsR R library used to access Uber’s H3 spatial index, [https://h3geo.org/](https://h3geo.org/), can be found here [https://github.com/obrl-soil/h3jsr](https://github.com/obrl-soil/h3jsr). The rhdx R library used to download Meta’s High Resolution Settlement Layer files from humdata.org can be found here [https://github.com/dickoa/rhdx](https://github.com/dickoa/rhdx).
