# Supplementary material for: What is rural? Examining the relationship between human populations and their inter-connectedness in the context of communicable disease transmission
Source: Int J Health Geogr. 2026 Mar 14;25:24. doi: 10.1186/s12942-026-00456-8 (PMC13104494; doi:10.1186/s12942-026-00456-8)
Supplement: Supplementary file 1 — Additional File 1 [file 12942_2026_456_MOESM1_ESM.pdf]

Additional File 1

What is Rural? Examining the relationship between human populations and their inter-connectedness in the context of communicable disease transmission

Authors

Cassandra Boutelle, Patrick Corbett, Andrew Gibson, Frederic Lohr, Catherine Swedberg, Jesse Blanton, Ryan Wallace

Alternative road data sources

The STARC methodology is built on the foundational idea that connectedness impacts disease transmission, and different road types facilitate different levels of connectedness. A road network file with a hierarchical classification of road segments is necessary to achieve this classification. OpenStreetMap (OSM) was selected for use in this paper, and it is the default source for obtaining road network files in the repository code. A summary of potential road sources is included in the table below.

**Data Source**	**Benefits**	**Drawbacks**
OpenStreetMap (OSM)	Most complete global road network; frequently updated; open license; transparent tagging schema	Variable data density in some regions; occasional inconsistencies in fclass labels
GRIP (Global Roads Inventory Project)	Harmonizes many (supra-)national datasets	Last updated 2018
gROADS (CIESIN / SEDAC)	Publicly available; some coverage in low- and middle-income countries	Sparse updates; limited tertiary/local roads
UNOSAT Road Layers (UNITAR)	High-quality in humanitarian response zones	Patchy global coverage; no unified global schema; infrequent updates
Proprietary road sources (e.g., Google Maps, HERE, TomTom)	Highly complete geometry; frequent updates	Cannot download or redistribute; no transparent metadata
National Government Road Datasets	Authoritative within-country; strong metadata in many high-income countries	No global coverage; incompatible schemas; restricted licensing

Of these sources, OSM has the most complete global coverage with a universal road hierarchy, so despite limitations of coverage and some inconsistencies in the fclass variable, OSM is the most viable option for the STARC methodology. If a more complete data source exists for a study area of interest, users may elect to implement STARC with another road data source, but one should take consideration for and make appropriate changes to the methodology to account for differences in road classification hierarchies.

Classification comparison

To both validate and highlight the additional benefits of STARC code classification, we conducted a comparison of STARC classification with the European Commission's degree of urbanization (DEGURBA) classification schema [1]. STARC and DEGURBA classification was conducted for four countries in West Africa (Guinea, Sierra Leone, Liberia, and Côte d'Ivoire).

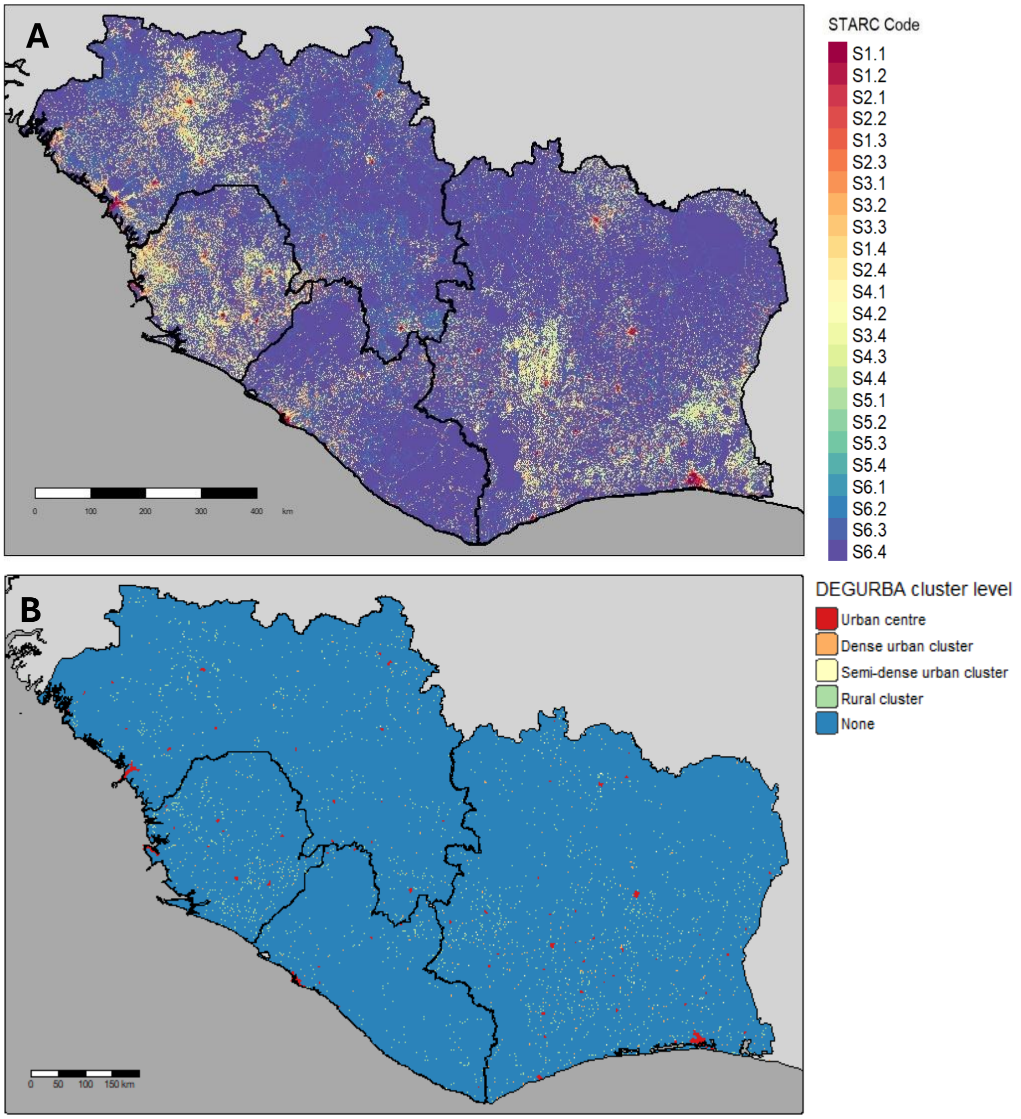

Visual comparison of STARC classifications (Panel A) and DEGURBA clusters (Panel B). Overall, higher STARC Codes (S1.y and S2.y) tend to overlap with DEGURBA Urban centres. STARC appears to highlight larger areas of land with low, but not no, population density that are not included in DEGURBA clusters.

STARC classification was assessed by calculating the proportion of land area in each DEGURBA population cluster level by STARC code. Analysis was conducted in R programming language version 4.0.3 using libraries such as tidyverse, sf, and raster [2-5]. DEGURBA clusters were intersected with each level of STARC code, and the area of intersection was divided by the total area of clusters of that DEGURBA cluster level. The results from each country were averaged and are summarized in the table below.

		STARC Code																							
		S1.1	S1.2	S1.3	S1.4	S2.1	S2.2	S2.3	S2.4	S3.1	S3.2	S3.3	S3.4	S4.1	S4.2	S4.3	S4.4	S5.1	S5.2	S5.3	S5.4	S6.1	S6.2	S6.3	S6.4
DEGURBA cluster level	Urban centre	39.4%	0.2%	0.0%	0.0%	27.6%	0.2%	0.1%	0.0%	25.7%	0.1%	0.1%	0.0%	4.2%	0.0%	0.2%	0.0%	0.7%	0.0%	0.3%	0.3%	0.0%	0.0%	0.1%	0.7%
	Dense urban cluster	14.2%	5.1%	1.8%	0.0%	26.7%	9.2%	3.0%	0.0%	12.5%	7.9%	1.9%	0.1%	5.2%	4.6%	2.0%	0.2%	1.0%	0.8%	0.9%	0.8%	0.0%	0.2%	0.7%	1.0%
	Semi-dense urban cluster	2.0%	2.0%	2.3%	0.0%	15.7%	17.4%	7.5%	0.2%	6.5%	8.0%	4.9%	0.2%	6.3%	8.6%	4.3%	0.4%	1.5%	2.2%	2.4%	1.9%	0.1%	0.2%	2.6%	2.7%
	Rural cluster	0.1%	0.3%	0.2%	0.0%	4.9%	10.7%	11.9%	1.3%	3.8%	7.3%	9.6%	1.4%	2.2%	7.1%	11.7%	1.1%	0.7%	2.7%	6.1%	3.2%	0.2%	1.0%	4.9%	7.4%

Average proportion of land area in Guinea, Sierra Leone, Liberia, and Côte d'Ivoire by STARC and DEGURBA cluster classification. Green shows where the classification schemas are most in agreement.

This table shows a loose agreement between STARC codes and DEGURBA cluster levels, especially as pertains to the human population density classification. As the DEGURBA cluster level decreases, the STARC level with the highest proportion of intersection decreases (i.e., the highest proportion of intersection for Urban centre is S1.1, while the highest proportion of intersection for Dense urban cluster is S2.1). These results also highlight the additional benefit of STARC's incorporation of road connectivity levels. As DEGURBA cluster levels decrease, the proportion of intersection becomes more spread across STARC road connectivity levels (e.g., Semi-dense urban cluster level has fairly even proportion of intersection across S2.1-S2.3), indicating that those cluster levels contain areas of land with various levels of connectivity. STARC is built on the foundational idea that road networks have the potential to facilitate spread of infectious disease. The inclusion of road connectivity in STARC's classification schema is uniquely suited for the STARC disease hotspot and cluster analysis, since it allows for the specification variables based on road connectivity.

References

1. European Commission, and Statistical Office of the European Union, *Applying the Degree of Urbanisation — A methodological manual to define cities, towns and rural areas for international comparisons — 2021 edition*. Publications Office of the European Union, 2021. ISBN 978-92-76-20306-3 doi:10.2785/706535
2. Wickham H, A.M., Bryan J, Chang W, McGowan LD, François R, Grolemund G, Hayes A, Henry L, Hester J, Kuhn M, Pedersen TL, Miller E, Bache and M.K. SM, Ooms J, Robinson D, Seidel DP, Spinu V, Takahashi K, Vaughan D, Wilke C, Woo K, Yutani H, *Welcome to the tidyverse.* Journal of Open Source Software, 2019. **4**(43): p. 1686.
3. Pebesma, E., & Bivand, R., *Spatial Data Science: With applications in R*. 2023: Chapman and Hall/CRC.
4. Pebesma, E., *Simple Features for R: Standardized Support for Spatial Vector Data.* The R Journal, 2018. **10**(1): p. 439-446.
5. Hijmans, R.J., *raster: Geographic Data Analysis and Modeling*. 2023.
