# Supplementary figures and images for: What is rural? Examining the relationship between human populations and their inter-connectedness in the context of communicable disease transmission
Source: Int J Health Geogr. 2026 Mar 14;25:24. doi: 10.1186/s12942-026-00456-8 (PMC13104494; doi:10.1186/s12942-026-00456-8)

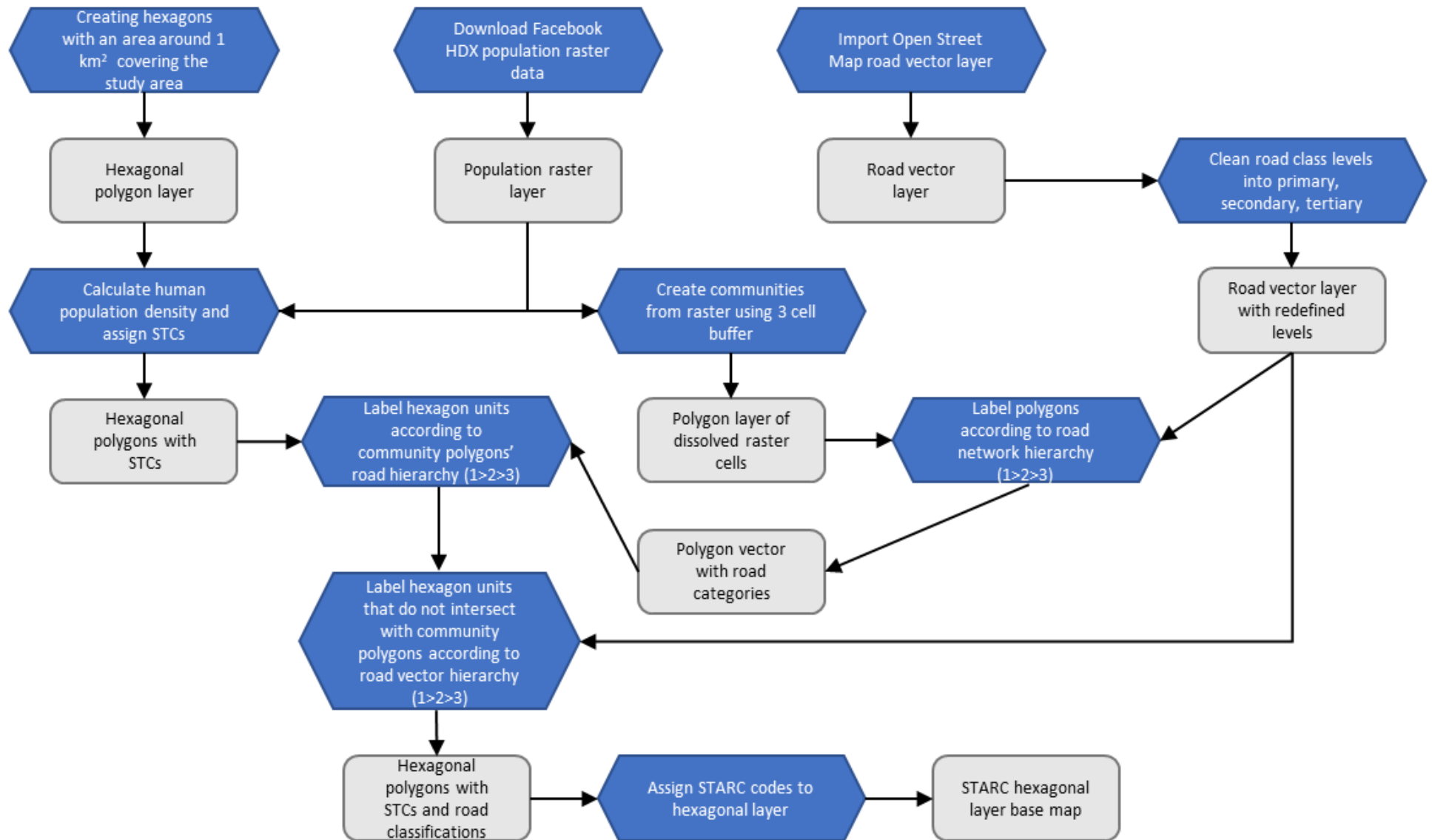

Supplement: Supplementary file 2 — Additional File 2 [file 12942_2026_456_MOESM2_ESM.pdf]

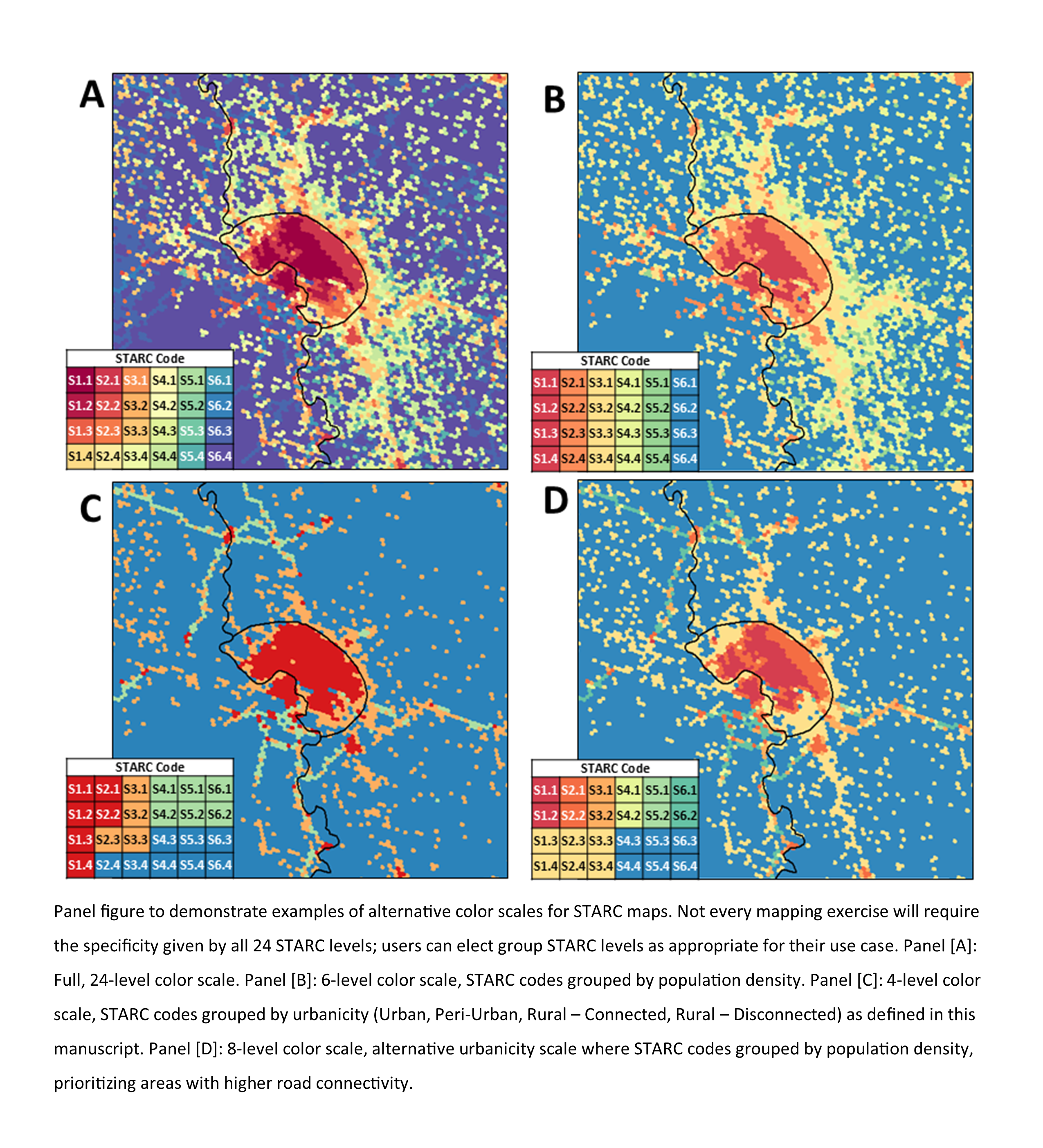

Supplement: Supplementary file 3 — Additional File 3 [file 12942_2026_456_MOESM3_ESM.png]

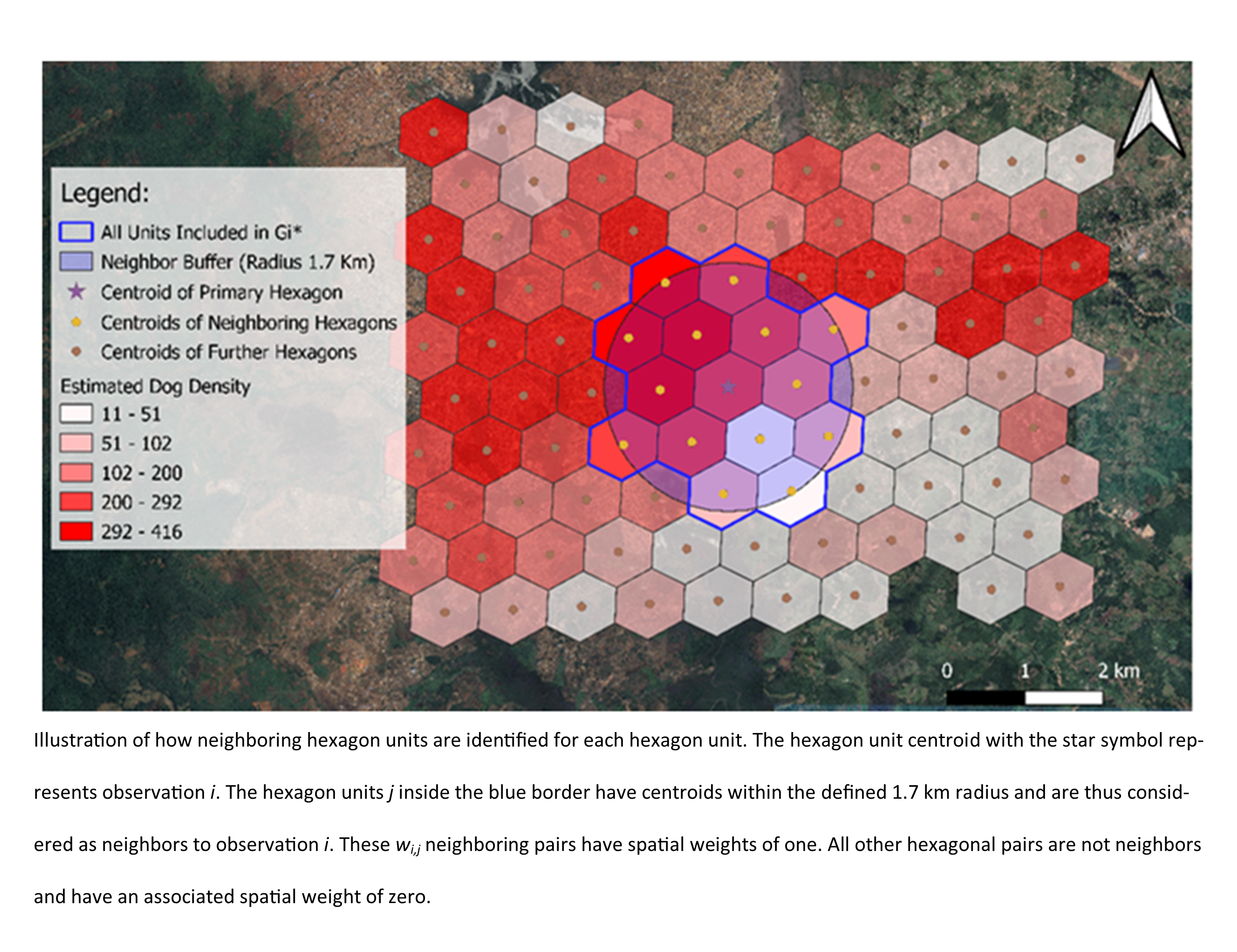

Supplement: Supplementary file 5 — Additional File 5 [file 12942_2026_456_MOESM5_ESM.png]
